# The Pluripotent Activities of Caffeic Acid Phenethyl Ester

**DOI:** 10.3390/molecules26051335

**Published:** 2021-03-02

**Authors:** Batoryna Olgierd, Żyła Kamila, Banyś Anna, Morawiec Emilia

**Affiliations:** 1 Department of Community Pharmacy, Faculty of Pharmaceutical Sciences in Sosnowiec, Medical University of Silesia in Katowice, 40-055 Katowice, Poland; kzyla@sum.edu.pl; 2 Department of Pharmaceutical Technology, Faculty of Pharmaceutical Sciences in Sosnowiec, Medical University of Silesia in Katowice, 40-055 Katowice, Poland; abanys@sum.edu.pl; 3 Department of Microbiology, Faculty of Medicine in Zabrze, University of Technology in Katowice, 40-555 Katowice, Poland; emilia.wojdas@gmail.com; 4 GynCentrum, Laboratory of Molecular Biology and Virology, 40-851 Katowice, Poland; 5 Department of Histology, Cytophysiology and Embryology in Zabrze, Faculty of Medicine in Zabrze, University of Technology in Katowice, 40-555 Katowice, Poland

**Keywords:** caffeic acid phenethyl ester (CAPE), propolis, inflammation, antioxidant, NF-κB

## Abstract

Caffeic acid phenethyl ester (CAPE) is a strong antioxidant extracted from honey bee-hive propolis. The mentioned compound, a well-known NF-κB inhibitor, has been used in traditional medicine as a potent anti-inflammatory agent. CAPE has a broad spectrum of biological properties including anti-viral, anti-bacterial, anti-cancer, immunomodulatory, and wound-healing activities. This review characterizes published data about CAPE biological properties and potential therapeutic applications, that can be used in various diseases.

## 1. Introduction

**Figure 1:**
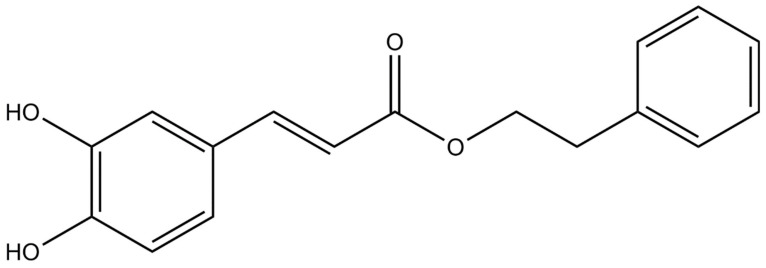
Chemical structure of caffeic acid phenethyl ester [3].

Propolis has been used in traditional medicine because of its wide spectrum of therapeutic benefits and its good historical safety profile. To date, more than 300 different compounds have been identified in propolis, including: aliphatic acids, esters, aromatic acids, fatty acids, carbohydrates, aldehydes, amino acids, ketones, chalcones, dihydrochalkones, terpenoids, vitamins and inorganic substances [1,2]. Caffeic acid phenethyl ester (CAPE) is one of the most promising bioactive ingredients found in propolis [3,4]. CAPE was identified as a component of propolis in 1987 and synthesized in 1988 at Columbia University [5]. Its molecular formula is C_17_H_16_O_4_ [3]. The structure of the compound is shown in Figure 1 [3]. CAPE is a polyphenol with hydroxyl groups in the catechol ring [6]. The presence of hydroxyl groups in the catechol ring is responsible for many of its biological activities [7]. CAPE has several biological properties including anti-inflammatory, antioxidant, anti-viral, anti-bacterial, immunomodulatory, anti-cancer and wound healing activities [5,8,9,10,11,12,13,14,15,16,17,18,19,20,21,22,23,24,25].

## 2. Biological Properties

### 2.1. Anti-Inflammatory Properties

CAPE has been described to conduct its anti-inflammatory activities by modulating different inflammatory pathways, including inhibition of the transcription factors NF-κB [25]. The NF-κB transcription factor plays a key role in a variety of physiological processes throughout the body, including immune responses, cell proliferation and inflammation [26]. NF-κB elicits its effects by promoting the transcription of many cytokines, chemokines, enzymes and antiapoptotic and cell growth factors [25].

NF-κB consists of homodimers and heterodimers from the Rel protein family, including p65/RelA, RelB, c-Rel, p50/p105 and p52/p100 [25,27,28]. Dysregulation of NF-κB and its dependent genes is associated with various pathological conditions including toxic/septic shock, graft versus host reaction, acute inflammatory conditions, acute phase response, viral replication, radiation damage, atherosclerosis, and cancer [29,30,31]. Activation of inactive NF-κB proteins present in the cytoplasm is induced by several factors, including pro-inflammatory cytokines (IL-1 and TNF-α), bacterial products and protein synthesis inhibitors [32]. NF-κB is a protein complex that controls DNA transcription and is the central regulator of cellular stress in all cell types in humans. NF-κB plays a key role in regulating the immune response to infection as well as in acute and chronic inflammation [33]. Therefore, compounds that can affect the activation of the NF-κB transcription factor have therapeutic potential because the activation of NF-κB is a critical step in the inflammatory cascade [34,35,36,37]. CAPE is potent and specific inhibitor of NF-κB activation and this may provide the molecular basis for its multiple immunomodulatory and anti-inflammatory activities [26,37,38].

The NF-κB proteins are held in the cytoplasm in an inactive state by an inhibitory subunit called IkBα. The phosphorylation of IkBα leads to the subsequent degradation of this protein and allows the translocation of NF-κB to the nucleus [38,39,40]. Many agents, including inflammatory cytokines (IL-1, IL-6 and TNF-α), enzymes (including nitric oxide synthase), adhesion molecules and acute-phase proteins, induce NF-κB [41,42,43]. CAPE has inhibitory effects on Helicobacter pylori-induced gastritis in Mongolian gerbils through the suppression of NF-κB activation and may thus have potential for prevention and therapy of Helicobacter pylori-associated gastric disorders [44]. CAPE suppressed Helicobacter pylori-induced NF-κB activation by inhibiting IκBα degradation and p65 phosphorylation in the gastric cancer cell line in a dose-dependent manner. Phosphorylation of IκBα acts as a trigger of IκB degradation, allowing the nuclear translocation of NFκB complex and activation of gene expression. Some studies have shown that CAPE inhibits NF-κB activation not by blocking the degradation of IkBα but by suppressing the interaction of NF-κB proteins with DNA [24,25,45,46,47,48,49,50].

### 2.2. Wound Repair

Non-healing wounds are a large and growing “interdisciplinary” clinical problem. Therefore, there is a continuing need to develop new agents that accelerate the healing of acute and chronic wounds and ulcers. Modern methods of wound care are increasingly based on apitherapy, in which standardized, pharmacologically active fractions obtained from bee products are used. One such substance is propolis characterized by a documented, broad spectrum of biological activities that are potentially used in wound healing, i.e., antiseptic, anti-inflammatory, astringent, anesthetic and antioxidant activity [51,52,53,54,55]. These biological effects of propolis are necessary to accelerate the wound healing process [51,54]. The main components responsible for the biological activity of propolis are flavonoids, phenolic acids and their esters, such as the phenolic ester of caffeic acid described in this review [56].

**Figure 2:**
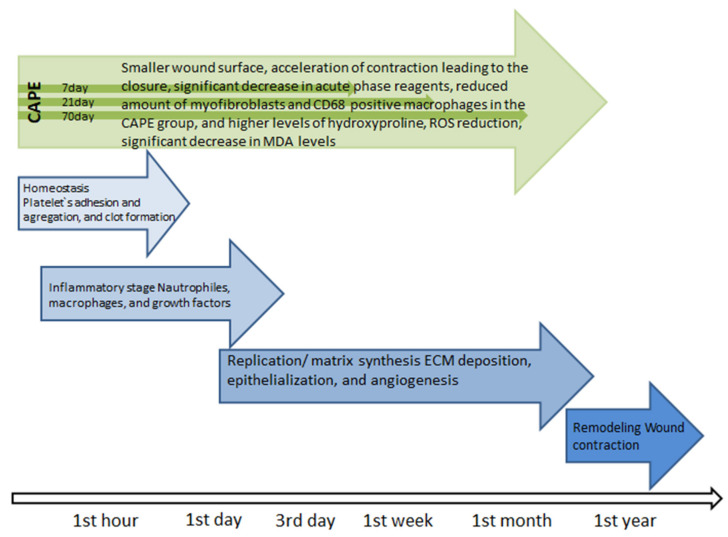
Healing stages [57].

Wound healing is the physiological response of the body that begins immediately after the appearance of the injury (Figure 2) [57]. The first stage hemostasis begins with narrowing of the damaged vessels [58]. Platelets are subject to adhesion, aggregation and activation, which leads to the formation of a blood clot that protects the structural integrity of the vessels and provides a temporary “scaffolding” enabling the formation of a temporary matrix in the wound bed. At the same time, cytokines, growth factors which interact with ECM components, are released into the wound bed, which initiates the repair process, preparing the damage bed for the next stage of the healing process, i.e., inflammation [57,59]. The inflammatory phase, which is initiated by neutrophils and develops under the influence of macrophages, is associated with cleaning the wound bed from bacteria and dead tissue debris, as well as releasing soluble mediators such as pro-inflammatory cytokines (IL-1, IL- 6, IL-8 and TNF-α) and growth factors (PDGF, TGF-α, TGF-β, IGF-1 and FGF) responsible for the recruitment and activation of fibroblasts [57]. Lack of neutrophils, as well as a reduced number of macrophages in the wound environment indicate that the inflammatory phase is coming to an end and the proliferation phase begins [57]. After the hemostasis and inflammation phase, lasting 2–3 days, the process of rebuilding the damaged tissue intensifies [57]. During this time, the number of cells in the wound bed increases, which is associated with the migration and proliferation of fibroblasts and endothelial cells, as well as keratinocytes, which secrete a number of mediators that stimulate ECM biosynthesis, promoting epithelization and angiogenesis [57]. The temporary matrix, formed mainly from the fibrin and fibronectin networks, is replaced by a collagen matrix enriched in proteoglycans, glycosaminoglycans and non-collagen glycoproteins, which leads to the restoration of the structure and function of the proper tissue [57]. The key cells of the ECM biosynthesis phase are fibroblasts, which migrate to the site of damage within 48–72 h from the onset of injury [57], starting the synthesis of ECM components and the creation of new connective tissue with a characteristic “granular” appearance interlaced with many capillaries [57]. This tissue appears around the fourth day after injury [59,60]. It consists of collagen (mainly type I and III), elastin, proteoglycans, glycosaminoglycans and non-collagen proteins [57]. The consequence of ECM biosynthesis is epithelization. It is a multi-phase process which involves reconstruction of the epithelium after injury [57]. The epithelization process is followed by an angiogenesis stage in which new blood vessels are formed [61]. This process restores blood circulation at the site of damage and prevents the development of ischemic necrosis, while stimulating the process of tissue repair. Remodeling is the last phase of the healing process during which the wound surface shrinks [57]. During this phase of the healing process, granulation tissue “matures” into a scar, which is accompanied by an increase in the mechanical strength of the newly formed tissue [57].

Studies on the treatment of thermal burns in rats conducted with the use of CAPE showed macroscopically 7, 21 and 70 days after the burn of a smaller wound surface and acceleration of contraction leading to the closure of the thermal damage to the skin in groups where CAPE was used compared to groups not supplied with the described compound. The positive effects of CAPE on the regeneration process of damaged skin in this study was also confirmed by biochemical analysis carried out 14 days after the burn. Analysis of myeloperoxidase activity showed a significant decrease in acute phase reagents. The nitric oxide determination showed a similar result. Tissue analysis 70 days after the burn also confirmed the long-term effect of CAPE, as it showed a reduced amount of myofibroblasts and CD68 positive macrophages in the CAPE group, and higher levels of hydroxyproline. These data show that the effect of CAPE on burn healing is still visible even after 70 days (CAPE administration stopped 14 days after the burn) [62]. Attention should be paid to some kind of modulation of proinflammatory mediator expression by CAPE used in wound healing. Studies in bedsores in mice have shown that the use of CAPE initially increased the expression of proinflammatory mediators (such as NOS2, TNF-α and NF-κB), infiltration of inflammatory cells, and oxidative damage (such as lipid peroxidation and peroxynitrite production). After 7 days, the expression of these parameters was reversed. This indicates the phenomenon of CAPE promoting early inflammatory response and associated oxidative stress as a short-term event, ultimately leading to accelerated healing of skin wounds and inhibition of inflammation and oxidative stress in the long term [19]. The process of treating damaged skin may hinder oxidative stress. CAPE has an antioxidant effect, thanks to which it can be widely used in the treatment of wounds of various origins, from minor skin abrasions to difficult-to-heal wounds such as burns, ulcers and pressure sores [63]. This is confirmed by studies on the effect of CAPE on the healing of skin wounds in rats, where a significant increase in the level of glutathione (GSH), which is an endogenous antioxidant and plays a key role in cellular defense in oxidative stress, has been shown in damaged CAPE-supplied tissues [63]. In the study, it was observed that GSH showed a progressive increase in the CAPE group, with significant differences on days 7 and 14, compared to the control group. CAPE, through its own antioxidant properties, can prevent GSH depletion. There was also a significant decrease in malondialdehyde (MDA) and superoxide dismutase activity in the CAPE group compared to the control group. Reactive oxygen species (ROS) are produced in response to skin damage and may cause cell damage by membrane lipid peroxidation, protein cross-linking and DNA breakdown. The study found a significant decrease in MDA levels in the CAPE group, especially on days 7 and 14 [63]. The multidirectional effect of CAPE in the form of free radical scavenging, anti-inflammatory and antimicrobial makes it an ideal candidate for the treatment of difficult-to-heal wounds of various origins.

### 2.3. Antidiabetic Properties: Obesity

CAPE can be useful in many pathological conditions caused by oxidative stress [63,64]. The increase in oxidative stress in diabetic patients is associated with a decrease in cellular antioxidant defense mechanisms. The transcription factor Nrf2 (nuclear factor-erythroid 2-related factor 2) is the main regulator of the body’s antioxidant response by affecting the expression of hundreds of genes that promote many antioxidant/detoxifying enzymes. A strategy based on the pharmacological regulation of Nrf2 may be a target in the treatment of metabolic disorders such as diabetes mellitus [65]. Hyperglycemia induces an increase in the level of reactive oxygen species (ROS) and nitrogen species (RNS), which cause oxidative/nitroso stress and, consequently, tissue damage. Studies show that inducible nitric oxide synthase/gamma-glutamylcysteine ligase (iNOS/GGCL) and dimethylaminohydrolase (DDAH) dysregulation may play a key role in oxidative stress mediated by high glucose levels, while heme oxygenase-1 inductors (HO-1), such as CAPE or its stronger derivatives, may be useful in diabetes and other oxidative stress-related conditions [65]. An in vitro study in rats with type 1 diabetes induced with streptozotocin showed that CAPE and its analogues were HO-1 inducers. Some of the compounds tested were stronger HO-1 inducers than CAPE. In particular, 2-(3,4-dihydroxyphenyl)-(2*E*)-2-propenoic acid (3,4-dimethoxyphenyl) ethyl ester (VP961) was the strongest. In addition, VP961 is the first known compound capable of directly activating HO-1 and simultaneously inducing protein expression of this enzyme [64,65]. Other studies conducted in rats towards the use of CAPE in diabetes therapy have shown that caffeic acid phenethyl ester inhibits the enzyme 5-lipoxygenase already at micromolar concentrations, and alleviates diabetic atherosclerotic symptoms, which are a major macrovascular complication of diabetes, increasing the risk of myocardial infarction and stroke. CAPE has also been shown to inhibit the increase in serum TNF-α levels, induce HO-1 aortic expression, and reduce collagen deposition. CAPE administration virtually abolished diabetes-induced atherosclerosis symptoms without affecting developed hyperglycemia. These results suggest that CAPE may be an important apitherapeutic agent protecting the vascular system during diabetes [66].

Obesity is associated with numerous clinical disorders, such as hypertension, hyperglycemia, insulin resistance, endothelial dysfunction, elevated triglycerides and high cholesterol [67]. Adipocytes, are one of the main cells that build adipose tissue, produce a variety of biologically active molecules, including interleukins, TNF-α, resistin, leptin, adiponectin, monocyte chemotactic protein (MCP-1), transforming growth factor (TGF-β), insulin-like growth factor (IGF-1) and C-reactive protein (CRP). Deregulation in the area of action of these factors leads to the general inflammation found in obesity [68]. CAPE has been shown to be effective in reducing acute inflammation induced by LPS in mature adipocytes derived from human ASC (Adipose Stem Cells) [67]. LPS is known to stimulate lipolysis. Excessive lipolysis contributes to the high level of circulating fatty acids and the development of insulin resistance-associated dyslipidemia seen in metabolic syndrome [69]. CAPE restores the function of adipocytes by increasing adiponectin and PPARγ (Peroxisome proliferator-activated receptor gamma), which leads to the reduction of pro-inflammatory factors. PPARγ is the main gene regulator responsible for the involvement of adipocytes, and its activation leads to the improvement of insulin sensitivity [70]. Adiponectin represents the insulin sensitizing, anti-inflammatory and anti-apoptotic hormone released by functional adipocytes. Obesity is also accompanied by oxidative stress, characterized by increased formation of reactive oxygen species (ROS). As mentioned before, CAPE is a compound with a well-documented strong antioxidant effect. A preventive effect of CAPE on oxidative stress was observed during the differentiation of 3T3-L1 cells into adipocytes by inhibiting the production of reactive oxygen species and increasing the activity of superoxide dismutase inside the tested 3T3-L1 cells [71].

### 2.4. Anti-Cancer Properties

The mechanism of CAPE anti-tumor activity is complex and omni-directional. CAPE can express an anti-cancer effect by inhibiting DNA synthesis [15,71], interruption of growth signal transmission [15,26], induction of apoptosis via an internal apoptotic pathway [15,72,73,74], and promoting anti-angiogenic effects [15,74,75]. The potential use of such broad mechanisms of CAPE anti-tumor activity in cancer chemotherapy can be considered in several ways. Separately, when CAPE is the main active compound in planned chemotherapy or as a supplement to chemotherapy, or when CAPE is an adjuvant strengthening the anti-cancer effect of another active compound used in chemotherapy, and as a chemopreventive agent, it protects normal cells against the cytotoxic effects of anti-cancer drugs [76].

#### 2.4.1. CAPE as an Adjuvant

Cancer chemotherapy requires the introduction of new solutions in the treatment regimen due to drug resistance, low effectiveness and many adverse effects of chemotherapeutic agents. Some tumors are internally resistant to many of the potent cytotoxic agents used in cancer therapy. Other cancers, initially sensitive, are not susceptible not only to the initially used therapeutic agents, but also to other drugs not used in the initial stages of therapy [77,78]. Combinations of drugs that interact with or complement each other with the anticipated therapeutic effect seem to be the right direction to conduct research to overcome problems associated with chemotherapy [15,70,79,80,81]. Modern medicine treats cancer using a combination of different treatment regimens. Therefore, substances of natural origin with documented anti-cancer activity have the potential as a complementary therapy in chemotherapy or radiation therapy. Many studies indicate the possibility of using CAPE as an adjuvant for a chemotherapeutic agent. CAPE treatment enhances the antitumor effect of cytostatic drugs, such as vinblastine, paclitacol, estramustine and docetaxel, used in the chemotherapy of prostate cancer [76,81,82]. Concomitant use of doxorubicin and cisplatin together with CAPE in gastric cancer has increased tumor sensitivity including chemo-resistant cancer cells, to cytostatics. CAPE increases the apoptotic cancer cell death induced by these two drugs by the accumulation of 4-hydroxy-2-nonenal (HNE), which is a highly reactive protein-bound aldehyde formed as a result of peroxidation by reactive oxygen species of membrane lipids [83]. CAPE inhibits HNE detoxifying enzymes (aldoketo reductase (1B1 and 1B10)) strongly expressed in gastric cancer cells, leading to HNE accumulation [84]. Reverse transcription-PCR analysis used in this mentioned study showed that CAPE treatment led to a reduction in the proteolytic activity of five proteasome subunits (PSMB1-PSMB5) and three immunoproteasome subunits (PSMB8-PSMB10) in doxorubicin-resistant gastric cancer cells. This means that CAPE can be used as a valuable adjuvant in chemotherapy for doxorubicin and cisplatin in gastric cancer. CAPE may find a similar application as an adjunct to treatment in chemotherapy of lung cancer, where it increases the sensitivity of cancer cells to doxorubicin [85]. CAPE also enhances 5-fluorouracil-induced inhibition of oral cancer cell growth [84]. 5-fluorouracil is widely used in head and neck cancers; it has a large spectrum of side effects including diarrhea, nausea, vomiting, mouth ulceration, lack of appetite, watery eyes, photophobia, metallic taste in the mouth during infusion, and a decrease in blood cell counts. Co-treatment of the TW2.6 cell line of the oral squamous cell carcinoma with CAPE and 5-fluorouracil showed additive inhibition of cell proliferation [86,87]. Due to the location of oral cancer, 5-fluorouracil is administered topically in the form of a cream or solution directly on skin lesions. Incorporation of CAPE into 5-fluorouracil topical therapy against oral cancer may reduce the amount and severity of side effects during chemotherapy. An additional advantage of using a lower concentration of the anti-cancer drug in addition to affecting the side effects is the reduction in the cost of therapy, which in the case of cancer chemotherapy is exceptionally large [86]. The combination of tamoxifen and CAPE applied against the human breast cancer cell line MCF-7 showed a synergistic cytotoxic effect manifested by significant activation of apoptotic mechanisms, along with a decrease in Bcl-2 protein and beclin-1 protein [88]. An additional benefit of the combination of CAPE + tamoxifen is the possibility, as in the case of 5-fluorouracil, to use a lower concentration of both drugs, which is particularly important due to the numerous side effects of anti-cancer drugs and the high cost of therapy. This has been confirmed by studies with the simultaneous use of tamoxifen and CAPE in breast cancer. Both tamoxifen and CAPE disrupt the antioxidant system in cancer cells by acting as pro-oxidants, stimulating apoptosis from excess reactive oxygen species. It should also be noted that only CAPE acts selectively on diseased cells, without adversely affecting normal cells [88].

#### 2.4.2. Chemoprevention

The interaction of anticancer drugs with substances of natural origin such as CAPE may be useful as support for cancer therapy in terms of chemoprevention of non-cancerous cells [86,88,89]. CAPE, in contrast to tamoxifen, proapoptotically affects only cancer cells, without affecting healthy, non-cancer cells. As a result, it does not have the side effects that are characteristic of anti-cancer drugs. The CAPE tamoxifen tandem prolonged the life of animals with Ehrlich tumor and caused a marked regression of tumor size and weight compared to animals treated with tamoxifen or CAPE only [88].

#### 2.4.3. Effect on Angiogenesis and Metastasis

Angiogenesis, the formation of new blood vessels, is an essential step in the progression of cancer, which allows the supply of nutrients and oxygen, as well as the removal of side products from cancer-changed tissues. In addition, newly formed blood vessels are the gateway to the expansion of cancer cells and the formation of metastases in other tissues and organs [90,91]. The regulation of tumor angiogenesis depends on the balance between angiogenic and anti-angiogenic factors produced within the pathological microenvironment. Disorders of this process lead to tumor angiogenesis. VEGF (vascular endothelial growth factor) acts as a strong angiogenic factor in pathological angiogenesis, it is secreted from tumor cells within the tumor, increasing its growth and contributing to metastasis. In addition, VEGF is necessary for the migration, permeability and proliferation of endothelial cells in the process of pathological angiogenesis. A significantly higher concentration of VEGF was observed in the regions of richly vascularized malignant tumors. Patients with elevated serum VEGF levels had a worse prognosis compared to patients with low VEGF levels. It is known that VEGF and its VEGFR-2 receptor, also called KDR, are required for the formation of new capillaries from pre-existing blood vessels in cancerous tissues. They play an important role in tumor progression, causing tumor growth, invasion and metastasis [92,93]. CAPE inhibits the VEGF-induced VEGFR-2 signaling pathway by suppressing VEGFR-2 tyrosine phosphorylation in endothelial cells. The inhibitory effects of CAPE on VEGF/VEGFR-2-dependent angiogenesis not only affect tumor growth but also endothelial cell activation [22]. Confirmation of the beneficial effects of CAPE on tumor mass and tumor size were studied in mice with Ehrlich tumor. The combined use of tamoxifen and CAPE resulted in the highest life expectancy of the mice and the lowest values of tumor size and mass [74]. CAPE controls tumor growth by stimulating angiostatic factors and inhibiting angiogenic factors whose expression deviates from physiological balance [94]. It has been suggested that blocking signaling by the NF-kB transcription factor inhibits angiogenesis and carcinogenicity in various types of cancer cells by suppressing VEGF expression [88,95,96]. CAPE, by inhibiting the NF-κB nuclear factor in human MCF-7 breast cancer cells, inhibits VEGF expression, and thus angiogenesis [88,94,96]. CAPE treatment reduces vascular endothelial growth factor (VEGF) secretion by inhibiting the ROS, PI3K and HIF-1α signaling pathways in human retinal pigment epithelial cells under hypoxia [86,97].

Tumor metastases include ECM degradation [86,98], which is regulated by matrix metalloproteinases (MMPs) [86,99]. The MMP gene is expressed in response to stimulation by several pro-inflammatory cytokines such as TNF-α, interleukin (IL) -1β and IL-6. MMP activity is regulated by several types of inhibitors, among which metalloproteinase tissue inhibitors (TIMPs) are the most important [100,101,102]. The balance between MMP and TIMP is responsible for controlling ECM protein degradation. MMP-2 and MMP-9 are the main enzymes involved in ECM degradation [103,104]. CAPE exerts an inhibitory effect on the expression and activity of MMP-2 by upregulating TIMP-2 and strongly reduces migration by reducing the focal phosphorylation of adhesive kinase (FAK) and the activation of its further p38 signaling molecules, mitogen activated protein kinases (p38 MAPK) and N-kinase final c-Jun (JNK) [105]. These data indicate that CAPE can potentially be used to prevent metastasis of oral cancer and that CAPE anti-tumor activity is at least partly regulated by the tumor microenvironment [86].

Both mRNA and COX-2 protein levels (present in inflamed sites) are strongly upregulated in OSCC (oral squamous cell carcinoma) [86,106] and high-risk precancerous lesions [87,107]. Increasing COX-2 regulation correlates with higher lymph node metastases, faster cell proliferative activity, and poorer survival rates in patients with oral cancer [88,108]. Treatment of CAPE 35–70 μM inhibits COX-2 activity and expression in human squamous cell carcinoma cells 1483 [86,109].

### 2.5. New Directions of Application

The therapeutic efficacy of CAPE was studied in rats with experimental endotoxin-induced periodontitis. Osteoclastic bone resorption is conjugated to an increase in RANKL, which causes destruction of follicular bone in periodontitis. To analyze follicular bone loss, RANKL activation was evaluated. Histological analyzes revealed lower RANKL levels in the CAPE treated group than in the saline group [110,111]. This result may be associated with the anti-osteoclastic effect of CAPE by suppressing RANKL-induced osteoclastogenesis, thereby inhibiting follicular bone loss. As a result, these findings confirmed that CAPE [110,112,113] improves bone healing, prevents RANKL-induced osteoclastogenesis, and can be used as a regenerative agent in bone resorption therapy.

CAPE demonstrated in dementia research an improvement in memory and prevention of memory impairment by enhancing antioxidant protection as a result of increased GSH (reduced glutathione) levels in rat brains. Reduced glutathione is the basic protection of the brain against oxidative stress and is used as a biomarker of oxidative stress [114].

## 3. Conclusions

The global demand for therapeutic alternatives in cancer therapy means that more effective and cheaper chemotherapeutic drugs are still being sought. Therefore, combinations of chemotherapeutic drugs with substances of natural origin with documented anti-cancer effects such as CAPE give hope for optimistic results. The combined use of CAPE together with anti-cancer drugs can be an important element in the design of a comprehensive anti-cancer therapy strategy, covering both the aspect of treating the cancer itself through the use of the synergistic cytotoxic effect of CAPE and anti-cancer drugs, as well as equally important in the chemoprevention of non-cancerous cells exposed to adverse side effects of anti-cancer drugs [115]. Table 1 presents a wide spectrum of CAPE effects.

Despite the enormous advantages of CAPE, there are several issues that may cause problems with the actual implementation of this compound in practical medicine. Due to the phenolic nature of CAPE, it exhibits a large spectrum of biological activities that can be used in various disease entities, but it has low solubility, and thus poor bioavailability, which significantly limits its effectiveness and requires the use of high doses [115]. Problems with solubility and bioavailability can be overcome by introducing modern drug delivery systems, e.g., using albumin nanoparticles, providing better therapeutic effects at a lower dose [116]. Another problem that may be significant is the allergenic properties of propolis. It has been reported that 1.2–6.6% of patients with dermatitis are sensitive to propolis. CAPE obtained from propolis could be a potential source of allergies, so that patients with allergic predisposition could be exposed to an increased risk of side effects, although this requires more careful examination in the future [117,118]. Difficult-to-heal wounds, such as burns, ulcers or pressure ulcers, remain an essentially unsolved medical problem, therefore there is a constant need to search for new effective solutions in this area. Standardized, pharmacologically active fractions obtained from bee products, such as propolis and one of its most biologically active ingredients, CAPE, are characterized by scientifically proven, multidirectional action in the field of wound healing of various origins, being a significant support for newly designed solutions in this process. This is confirmed by numerous scientific studies, including our authorship, describing their impact on the wound healing process. One of our newest studies confirming the reparative and regenerative properties of propolis, and thus also CAPE, is the use of electron paramagnetic resonance spectroscopy (EPR) to determine paramagnetic centers occurring in blood samples taken from experimental animals, after the application of therapy with an innovative biodegradable propolis-nanofiber dressing [119]. Paramagnetic centers and changes in free radicals indicate the beneficial effect of the innovative combination of nanofibers with a natural ingredient with a broad spectrum of action, i.e., propolis, on the process of burn regeneration. The multifaceted influence of propolis on changes in the pro-oxidative-antioxidant balance, the components of which play a fundamental role in the repair of tissue damage, indicates the high potential of antioxidant compounds, including propolis, in accelerating wound healing, including thermal burns [119]. The above-mentioned observations confirm our previous research, which showed that the concentration of free radicals in the matrix of wounds treated with propolis was much lower than in the case of wounds treated with a standard drug-silver sulfadiazine, which is associated with the creation of a favorable biochemical environment by propolis supporting the wound healing process [52,56].

The pleiotropic effect of the caffeic acid phenethyl ester gives a wide range of therapeutic possibilities in many disease entities, in which CAPE has not been used so far, e.g., dementia or bone resorption. Therefore, this active compound will remain the subject of much future research. Human research needs to be significantly expanded as most research on CAPE is based on animal and in vitro studies.

## Figures and Tables

**Table 1:** Reported CAPE effects.

Effect	Reference
**Anti-Inflammatory Properties**	
Cells and neurons protection in acute septic shock	Korish and Arafa., 2011 [9]
Corneal neovascularization	Totan et al., 2001 [14]
Inhibition of thrombin activity	Melzig and Henke., 2005 [16]
Slowing the progression of amyotrophic lateral sclerosis	Fontanilla et al., 2012 [18]
Improved solubility and anti-inflammatory effect through transglycosylation	Li et al., 2019 [24]
NF-κB pathway regulation	Natarajan et al., 1996 [26] Wang et al., 2010 [31] Linard et al., 2004 [32] Choi and Choi., 2008 [35] Orban et al., 2000 [36] Lee et al., 2010 [41] Jung et al., 2008 [43] Toyoda et al., 2009 [44] Bezerra et al., 2012 [46] Andrade-Silva et al., 2009 [49] Kızıldağ et al., 2019 [110] Tambuwala et al., 2019 [116]
Reduction in the development of atherosclerosis	Hishikawa et al., 2005 [30]
Protection of cerebellar granular neurons against neurotoxicity	Wei et al., 2008 [1]
Protective effect on hepatic ischemia/reperfusion injury	Saavedra-Lopes et al., 2008 [38]
Inhibition of nitric oxide synthase	Song et al., 2002 [45]
Anti-inflammatory effect on endotoxin-induced uveitis	Ylmaz et al., 2005 [50]
Inhibition of cyclooxygenase-2	Michaluart et al., 1999 [109]
Prevention of dementia	Kumar et al., 2017 [114]
Hypoxia-inducible transcription factor regulation	Bhargava et al. 2018 [7]
**Wound Repair**	
Regeneration of burns	Dos Santos et al., 2013 [19]
Healing pressure ulcers	Romana-Souza et al., 2018 [62]
Accelerating skin wound healing	Serarslan et al., 2007 [63]
Bone treatment	Kazancioglu et al., 2015 [112]
**Anti-Diabetic Properties**	
Anti-diabetic effect	Abduljawad et al., 2013 [42]
Protective effect in type 1 diabetes	Sorrenti et al., 2019 [65]
Alleviation of diabetic atherosclerosis	Hassan et al., 2014 [66]
**Obesity**	
Regulation of PPAR levels in adipocytes	Vanella et al., 2016 [67]
Inhibition of oxidative stress in adipocytes	Yasui et al., 2013 [71]
**Anti-Cancer Properties**	
Cytotoxic effect on cancer cells	Grunberger et al., 1998 [5] Watanabe et al., 2011 [8] Kabała-Dzik et al., 2017 [55]
Pro-apoptotic effect on cancer cells	Hung et al., 2003 [11] Jin et al., 2008 [37] Chen et al., 2008 [73] Gherman et al., 2016 [78]
Restoration of gap junctional intercellular communication	Na et al., 2000 [17]
Chemoprevention	Carrasco-Legleu et al., 2004 [47]
Inhibition of tumor promotion	Huang et al., 1996 [72] Imai et al., 2019 [23] Lin et al., 2012 [81] Kuo et al., 2015 [86] Chuu et al., 2012 [89] El-Refaei et al., 2010 [94]
Effect on the proteasome	Peng et al., 2012 [105]
Cytotoxic adjuvant	Matsunaga et al., 2019 [76] Tolba et al., 2013 [82] Sonoki et al., 2018 [85] Kuo et al., 2013 [87] Motawi et al., 2016 [88]
Inhibition of human aldo-keto reductase	Soda et al., 2012 [84]
**Effect on Angiogenesis**	
Inhibition of angiogenesis	Song et al., 2002 [12] Paeng et al., 2015 [97]
Inhibition of VEGFR-2 activation	Chung et al., 2013 [22]
Inhibition of platelet-derived growth factor	Roos et al., 2011 [74]

## Data Availability

Data sharing is not applicable. No new data were created or analyzed in this study.
